# Should oncologists trust cannabinoids?

**DOI:** 10.3389/fphar.2023.1211506

**Published:** 2023-07-13

**Authors:** Ioana Creanga-Murariu, Leontina Elena Filipiuc, Magda Cuciureanu, Bogdan-Ionel Tamba, Teodora Alexa-Stratulat

**Affiliations:** ^1^ Advanced Research and Development Center for Experimental Medicine (CEMEX), “Grigore T. Popa” University of Medicine and Pharmacy, Iași, Romania; ^2^ Oncology Department, “Grigore T. Popa” University of Medicine and Pharmacy, Iași, Romania; ^3^ Pharmacology Department, Clinical Pharmacology and Algesiology, “Grigore T. Popa” University of Medicine and Pharmacy, Iași, Romania

**Keywords:** cannabis, cannabinoids, cancer, oncology, pain, nausea and vomiting, anorexia and cachexia, anxiety and depression

## Abstract

Cannabis enjoyed a “golden age” as a medicinal product in the late 19th, early 20th century, but the increased risk of overdose and abuse led to its criminalization. However, the 21st century have witnessed a resurgence of interest and a large body of literature regarding the benefits of cannabinoids have emerged. As legalization and decriminalization have spread around the world, cancer patients are increasingly interested in the potential utility of cannabinoids. Although eager to discuss cannabis use with their oncologist, patients often find them to be reluctant, mainly because clinicians are still not convinced by the existing evidence-based data to guide their treatment plans. Physicians should prescribe cannabis only if a careful explanation can be provided and follow up response evaluation ensured, making it mandatory for them to be up to date with the positive and also negative aspects of the cannabis in the case of cancer patients. Consequently, this article aims to bring some clarifications to clinicians regarding the sometimes-confusing various nomenclature under which this plant is mentioned, current legislation and the existing evidence (both preclinical and clinical) for the utility of cannabinoids in cancer patients, for either palliation of the associated symptoms or even the potential antitumor effects that cannabinoids may have.

## 1 Introduction

A natural remedy known for millennia, the cannabis plant has tranquilizing, hypnotic and aphrodisiac effects, used since ancient times to treat various conditions. The root, was recommended for treating inflammation, gout, arthritis, fever, skin burns, infections, postpartum hemorrhage, gastrointestinal diseases and tumors (Ryz et al., 2017). Cannabis inflorescence and leaves have also been used to treat epilepsy, glaucoma, insomnia, and pain (Brunetti et al., 2020; Jin et al., 2020). In addition to its medicinal role, cannabis has also been used as food, due to its high content of fiber and oils that are very nutritious (Zuardi, 2006). At the beginning of the 20th century, cannabinoids were used in various conditions worldwide. However, their side effects and increased use as recreational drugs led to the criminalization of cannabis in the US in 1937, thus greatly impacting further research that could offer a modern scientific understanding of its medicinal potential (Victor et al., 2022). The first decades of the 21st century have witnessed a resurging interest (Walsh et al., 2017) and more literature regarding the benefits of cannabinoids in various diseases has become available; however, because “cannabis” is in fact an umbrella term for many drugs, the various nomenclature under which this plant is mentioned can sometimes be confusing and lead to hindrances in data reproducibility and direct cross-study comparisons.

The popular term “cannabis” refers in general to all products (herbal or synthetic) derived from the plants belonging to the Cannabis genus, with cannabinoids being several active classes of chemical compounds found in the plant. More than 110 natural compounds, called phytocannabinoids, have been identified and over 100 lipophilic molecules have been isolated within their structure. Δ9-tetrahydrocannabinol (Δ9-THC) and cannabidiol (CBD) are perhaps the best-known representatives. CBD was first extracted in 1940 (Adams et al., 1940) and its full chemical structure was unraveled in 1963 by Mechoulam and others (Mechoulam and ShvoHashish, 1963), and Δ9-THC followed shortly after, being first isolated and structurally elucidated by the same team in 1964 (Gaoni and Mechoulam, 1964).

The term “herbal cannabis” commonly refers to the harvested dried female flowering tops of *Cannabis Sativa L*, which contain the highest concentrations of natural cannabinoids, mainly Δ9-THC, CBD, cannabigerol, cannabichromene, cannabidivarin and tetrahydrocannabivarin (Lewis et al., 2017; Odieka et al., 2022). Each strain of cannabis plant can have variations in the concentration of the substances within and also contains over 500 other chemical compounds such as cannabinoid phenols, non-cannabinoid phenols, alcohols, aldehydes, n-alkanes, alkaloids, flavonoids, terpenoids, wax esters and steroids, which in turn may modulate the effect of cannabinoids (the “entourage effect”) (Lewis et al., 2017; Odieka et al., 2022). Over time, growers have developed and selected specific cannabis strains due either to their high Δ9-THC content (greater psychoactive effect, source for marijuana and hashish), their high CBD content (most often used for medicinal marijuana) or their high fiber and oil content (hemp) (Hilderbrand, 2018), which lead to the great variability of legal and illegal cannabis-based produce available today.

Different proportions of major active ingredients along with smaller proportions of minor components, that enhance the effects of major phytocannabinoids, of cannabis offer different effects, so they can be used in different conditions. For example, in anorexia, nausea and vomiting it is recommended to use a product with a content of THC higher than CBD (THC > CBD); for insomnia and pain, approximately equal proportions of THC and CBD (THC ≅ CBD); and for anxiety and depression a content of THC lower than CBD (THC < CBD) (Brunetti et al., 2020).

The identification of the first endogenous cannabinoids and cannabinoid ligands (cannabinoid receptor 1 and 2—CB1R and CB2R) (Shahbazi et al., 2020) led to the development of so-called “cannabinoid probes” initially used for better characterizing the endocannabinoid system. Thus, synthetic cannabinoids (SCs) were created. When compared to phytocannabinoids, SCs have significantly more affinity–up to 4 times higher for CB1R and 10 times higher for CB2R and, as a consequence, may have greater psychoactive effects (Bukke et al., 2021). As such, SCs quickly entered the illegal drug market and were sold as recreational drugs. Their popularity has likely been enhanced by the lack of detection in typical urine drug screens for THC (Schneir et al., 2011). Most SC’s are sprayed onto herbal substances so they can be smoked or sold as liquids to be vaporized and inhaled (Tamba et al., 2020). This has led to a significant increase in cannabis-related hospital admissions, especially since acute, severe or unpredictable side effects have a higher incidence in SC users than in natural cannabinoids users (Forrester et al., 2012).

Another important challenge for the progress of cannabis research is the immense variability of both natural and synthetic cannabinoids. Each cannabis plant has a vast assortment of active compounds that vary in composition, concentration and ratio with environmental factors, genetic background and even morpho-spatial position of the plant (Danziger and Bernstein, 2021). Unfortunately, there are only few rigorous studies assessing the effect of plant architecture modulation treatments and other methods for obtaining standardized cannabis cultures (Kocjan Ačko et al., 2019). As such, preclinical studies usually use SCs or purified cannabis oil and clinical studies are usually forced to use the few cannabis-based produce that are already approved and standardized. Most countries have efficient regulatory bodies for cannabis based medicinal products that have undergone a regular marketing authorization process, which ensure the compliance with the Good Manufacturing Practices (GMP), however some products go through more simplified authorization processes, or even exempted from specific regulatory authorizations, thus no guarantee that these aspects are met (Seddon and Floodgate, 2020; de Souza et al., 2022). Illicit SCs have no production standards whatsoever, so two drugs sold under the same street name can in fact have completely different doses, compositions or can contain poisonous or carcinogenic compounds.

Despite having enjoyed a “golden age” as a medicinal product in the late 19th, early 20th century, cannabis began being replaced by other drugs and its use progressively restricted mainly due to the increased risk of overdose and abuse (Crocq, 2020). In 1961, the United Nations Single Convention on Narcotic Drugs classified cannabis as belonging to 2 categories: schedule I, substances that are highly addictive and liable to abuse, and schedule IV, substances that are highly addictive, liable to abuse and lack therapeutic value. This meant that cannabis use for all purposes, including medical use, was to be prohibited (Bayer and Ghodse, 1999). However, in time, a significant body of data that outlined the therapeutic effects of cannabis has been published. In consequence, in December 2020, the United Nations’ Commission for Narcotic Drugs recommendations were revised, concluding that cannabis should be listed only in schedule I of the system, because of its demonstrated therapeutic potential (Mahabir et al., 2020). As a consequence, different countries have adopted more permissive laws regarding cannabis use for either medicinal or recreational purpose. However, the legalization and decriminalization of cannabis are two terms that have different meaning and should not be confused. Legalization allows the production and sale of either recreational or medical marijuana and marijuana-infused products. Decriminalization of cannabis means that while its use is still illegal, it is rather considered a minor infraction with less severe punitive consequences.

**FIGURE 1:**
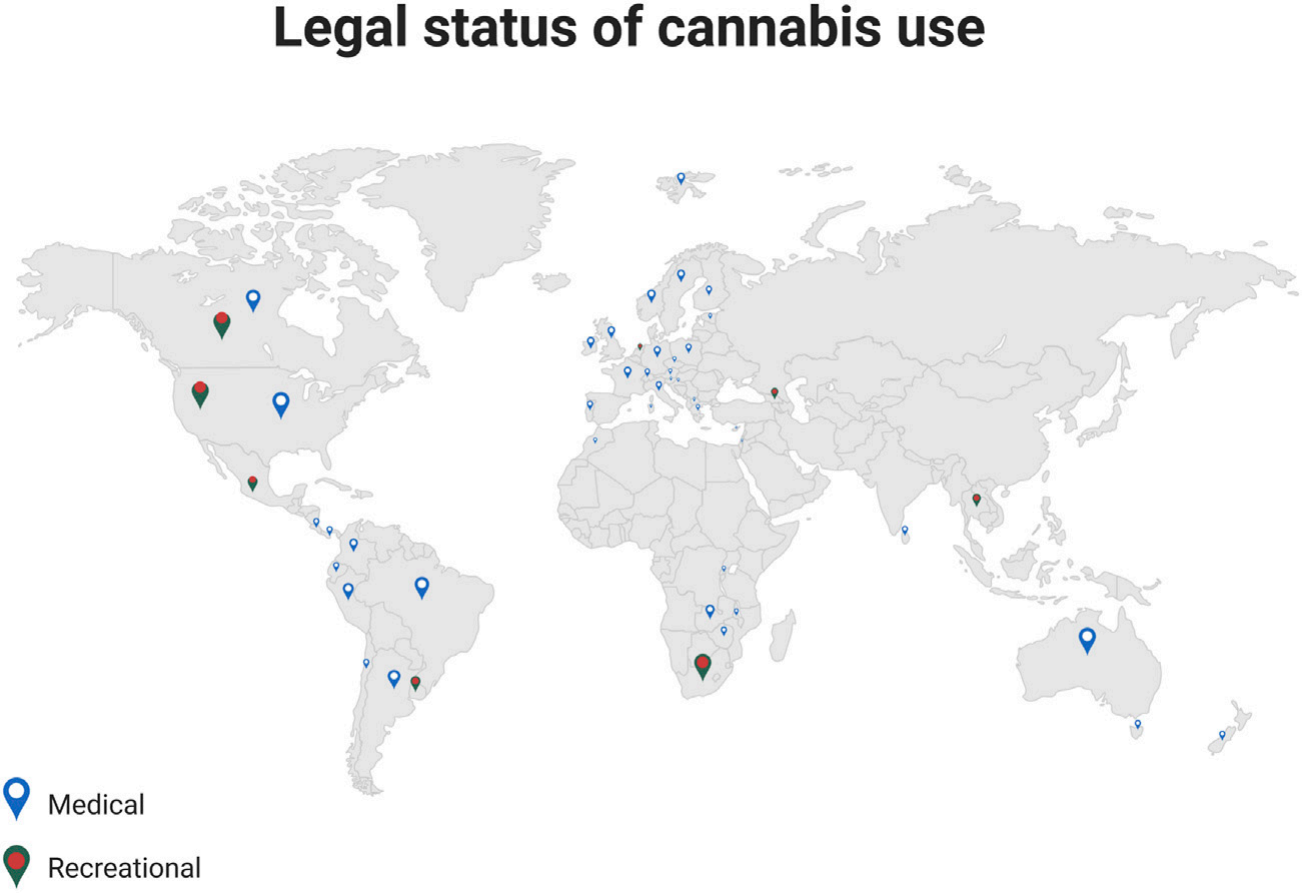
Legal status of cannabis use. The illustration provides information on a worldwide scale, regarding the current regulations for prescribing medical cannabis, and for the recreational use (Adapted from Bio Render).

The first country to legalize Cannabis use for medical and recreational use was Uruguay in 2013 (Seddon and Floodgate, 2020). Several European states also revised their legislation for medical cannabis use in recent years (Figure 1):

In the United States, 18 states have legalized, so far, the use of Cannabis for medicinal purposes (Mahabir et al., 2020). Despite the fact that the recreational use is still illegal in most of the states, some 48 million people, or about 18% of Americans, have used Cannabis at least once (Compton et al., 2016), with cannabis-related illicit sales reaching $17.5 billion in 2020 (Yakowicz, 2023).

Although recent years have seen a trend towards decriminalizing marijuana use, the Foods and Drugs Administration (FDA) and The European Medicines Agency (EMA) have not yet approved the use of the cannabis plant for any medical purpose. However, both the FDA and EMA have approved the use of drugs that contain individual cannabinoids (Whiting et al., 2015). Some examples include Epidiolex, a purified form of CBD used for the treatment of seizures associated with Lennox-Gastaut syndrome or Dravet syndrome (Sekar and Pack, 2019) (FDA and EMA-approved) or Dronabinol (FDA-approved)—a synthetic THC used as an appetite stimulant in AIDS-related cachexia (Badowski and Yanful, 2018) and for alleviating chemotherapy-induced nausea and vomit in cancer patients (May and Glode, 2016). Another area of interest is the treatment of post-traumatic stress disorder (PTSD) in military personnel with THC or nabilone (Sholler et al., 2020). Nabilone (a synthetic analogue of THC, Cesamet® or Canemes®), ten times more potent than natural THC, is FDA-approved for the treatment of nausea and vomiting for patients receiving chemotherapy who have failed to respond to conventional antiemetics (Vinette et al., 2022). Recently, the effectiveness of nabilone has been assessed in the treatment of neuropathic and chronic pain, and in spasticity related to multiple sclerosis (Manera and Bertini, 2021). Nabiximols (Sativex®), available as an oral spray, that contains both THC and CBD is approved in the United Kingdom, Germany, and Switzerland for multiple sclerosis (MS)-related spasticity and in Canada for pain associated with MS and cancer (Keating, 2017).

Marketing authorization of cannabinoids in Europe can be obtained either with the centralized procedure, via the EMA, or through the non-centralized route where medication may be authorized in individual EU countries through the national competent authorities. Figure 1 shows the countries in which medical cannabis consumption is permitted. Nabiximols is available in most EU states, and even if Dronabinol and Nabilone are less widespread, they are still available in around one third of EU.

Along with the renewed interest in cannabinoids as drugs for chronic neurological disorders, an increased focus on cannabis and its potential applications in oncology has also resurged. Cannabinoids are frequently used both on- and off-label by cancer patients and several multicenter studies have suggested a 18%–40% prevalence of use in this populational group (Abrams, 2022).

However, oncologists seem to be reluctant to follow this trend. A 2018-study assessing 400 oncologists’ perceptions on the issue reported that 70% of them think they have concerning gaps in data regarding cannabinoid use in cancer patients, even though most of them (80%) reported conducting discussions with patients. This reluctance could be because physicians consider that available data about cannabinoids as alternative therapy is contradictory and difficult to evaluate given the sparse randomized controlled trial data (Braun et al., 2018). The same attitude was seen in a recent systematic review, where 21 different studies conducted in United States, Canada, Europe, Australia, and Israel, were included. The lack of knowledge of its clinical effects varied between clinicians, however the physicians experienced in prescribing medical cannabis were less worried about the adverse effects (Rønne et al., 2021). Additionally, the lack of standardization of most cannabinoids used outside standard recommendations are a major drawback for large database analysis, thus hindering data reproducibility and the possibility of identifying clinical benefits.

The motivation of the present article is that a physician should prescribe cannabis only if a careful explanation can be provided, and a follow up response evaluation, ensured. Oncologists should be up to date with the positive and negative aspects of the administration of cannabis in cancer patients as both prevalence and demand has increased in the past years. A better understanding of cannabinoids’ pharmacokinetics, side effects and efficacy profiles as well as their formulations is thus required.

## 2 Palliation of cancer-associated symptoms

The unique qualities of each cannabis variety or chemovar are the result of distinct concentrations of numerous classes of bioactive molecules, most notably, cannabinoids, terpenoids and flavonoids. As such, cannabinoids have various effects that depend on the dose and the compound used. The plethora of effects can also be explained by the fact that cannabinoids bind to both CB1R and CB2R, and non-cannabinoid receptors, such as the adrenergic receptors, vanilloid receptor 1 (TVRP1), transient receptor ankyrin 1 potential (TRPA1), peroxisome proliferator-activated receptor-gamma or glitazone receptor (PPAR-γ), G55 protein-coupled receptor (GPR55), and nuclear receptor (NRs) (Huang et al., 2020). This broader view of ligands and enzymes involved in the endocannabinoid system led to the concept of the endocannabinoidome, which encompasses hundreds of lipid mediators and tens of enzymes and molecular targets (Huestis et al., 2019; Pryimak et al., 2021; Hinz and Ramer, 2022). As such, the potential effect of cannabinoids on the most prominent cancer-associated symptoms, such as pain, nausea, vomiting, cachexia, anorexia, depression or anxiety has been investigated in several clinical and preclinical studies.

### 2.1 Pain

Pain is an unpleasant sensory and emotional experience associated with actual or potential tissue damage, as defined by the International Association for the Study of Pain (IASP) (Raja et al., 2020). In cancer patients, pain is reported as one of the most distressing aspects of their condition and has been linked to numerous physical and mental ailments that contribute to high healthcare costs and loss of productivity (Stoorvogel et al., 2022). Available data indicate that chronic pain generates considerable pressure, being one of the most common reasons adults seek medical care (Rodriguez et al., 2019), with an estimated annual cost of more than $635 billion (Casey and Vaughan, 2018). Of note, current findings indicate that the burden of chronic pain in cancer patients is particularly alarming, with prevalence rates of 39.3% after curative treatment; 55.0% during anticancer treatment; and 66.4% in advanced, metastatic, or terminal disease (van den Beuken-van Everdingen et al., 2016). Moreover, more than one-third of cancer patients do not receive analgesic treatment proportional to or adequate for the intensity of their pain (Gress et al., 2020).

Neuropathic pain accounts for almost 40% of cancer pain cases (Oosterling et al., 2016). It is attributable to the cancer *per se* and/or because of treatment-related events, including chemotherapy-induced peripheral neuropathy (CIPN), post radiation plexopathies, and surgery for tumor resection (Haroun et al., 2022). Also, current treatment options for neuropathic pain are often ineffective, usually requiring association of several classes of drugs (Kachrani et al., 2020).

Opioids have been the mainstay of severe cancer pain treatment in the past decades. However, it is increasingly clear that these drugs should be carefully prescribed due to side effects and tendency to create dependence (Wang et al., 2021). Furthermore, the number of deaths secondary to opioid overdose is on the rise, with more than 65% of drug‐overdose deaths involving at least one opioid, most often morphine or fentanyl (Saloner et al., 2018). Last, but not least, some types of chronic pain are refractory to opioids, requiring invasive techniques that are not always accepted by the patient or anesthetics (Fallon et al., 2018), thus revealing the need for new or improved analgesic drugs.

Recent data suggests that patients are increasingly using cannabis as a substitute for prescription opioids, with various reported outcomes (Casey and Vaughan, 2018). Other studies have focused on the potential synergistic effects of opioids and cannabinoids, the so-called “opioid-sparing effect” (Nielsen et al., 2022), which could address the opioid epidemic by significantly decreasing the analgesic doses required for refractory cancer-related pain.

a) Cannabinoids mechanisms on pain

The effect of exogenous cannabinoids on different types of acute and chronic pain depends on the drug’s interaction with the endocannabinoid system, whose components are expressed almost ubiquitously throughout the nociceptive pathways (Finn et al., 2021). The activation of CB1R from primary afferent neurons, dorsal horn of the spinal cord and brain regions involved in pain processing is associated with a decrease in neuronal excitability and a dampening of neurotransmission (Brown and Farquhar-Smith, 2018). The activation of CB2R, mostly expressed on immune cells, has a plethora of suppressive effects including reduced cytokine release and decreased antigen presentation (Eisenstein and Meissler, 2015), which is extremely relevant for several physiological processes that can modulate pain perception, such as mood, stress response, immune and inflammatory response and neurotransmission (Mangal et al., 2021). Additionally, CB2R have been occasionally identified on neurons, especially after injury, thus making CB2 a potential target in neuropathic pain (Brown and Farquhar-Smith, 2018). Both endogenous and exogenous cannabinoids bind to one or both CB receptors with different affinity, which explains their different effects. For example, both 2-Arachidonoylglycerol (2-AG) and N-arachidonoylethanolamine (AEA), the best-known endogenous cannabinoids, and Δ9-THC are CB1R and CB2R agonists, but with different affinities, whereas CBD is a CB1R and CB2R antagonist. Synthetic cannabinoids are usually more specific, binding to either CB1R or CB2R; examples include AM1710 (CB2 agonist), AM630 (CB2 antagonist) and AM251 (CB1 antagonist) (Rahn et al., 2014; Blake et al., 2017). Additionally, the endocannabinoid network also contains G protein-coupled receptors, members of the transient receptor potential cation channel subfamily, 5-hydroxytryptamine receptor 1A, 2A, and 3A, peroxisome proliferator-activated receptor gamma receptors and others, all of which modulate pain or pain-related processes (Balachandran et al., 2021; Lord et al., 2022). Last, but not least, studies have identified changes in endocannabinoid levels after exposure to neuropathic and nociceptive stimuli, suggesting a dysregulation of the system could be involved in several types of chronic pain (Masocha, 2018). Although the understanding of this system has greatly advanced in recent years, there are still a lot of controversies surrounding the exact mechanism by which cannabinoids modulate different types of pain, which, when coupled with the large variety and heterogenicity of the investigated compounds, leads to mostly contradicting results in observational clinical studies.

b) Cancer pain-preclinical and clinical data

There is a broad spectrum of pain models in which cannabinoids have been tested in the past decades, mostly with good results, especially in chronic neuropathic and inflammatory pain, which have a high prevalence in cancer. Interestingly, a study on a murine model of cancer pain assessed synthetic CB1R and CB2R agonists individually and in combination and concluded that binding of any CB receptor is associated with pain alleviation comparable to that of morphine and the co-administration of the two agonists has synergistical effects (Khasabova et al., 2011), which is why several different natural and synthetic CB1R and CB2R agonists have been investigated in this setting. Intraperitoneal administration of WIN 55212-2, was shown to alleviate pain in both carrageenan-evoked and tumor-evoked hyperalgesia in a time and dose-dependent manner, although the authors noted that the effect on inflammatory pain was more potent than on cancer pain (Kehl et al., 2003). In a model of cancer-induced allodynia, systemic administration of WIN 55212-2, ACEA (CB1R selective) or AM1241 (CB2 selective) significantly decreased pain (Saghafi et al., 2011). More recently, MJN110 (a monoacylglycerol lipase inhibitor) administration alleviated cancer-induced bone pain in an animal model, most likely by increasing the endogenous cannabinoid concentration (Thompson et al., 2020). In the clinical setting, two trials from the 80’s performed by Noyes et al. compared THC to codeine (Noyes et al., 1975a; Noyes et al., 1975b) with good outcomes despite notable side-effects at high doses. Several other subsequent clinical trials have had conflicting results, most likely due to small sample size, varying types of cannabinoids used and different endpoints analyzed. Several systematic reviews and meta-analysis have focused on gathering these data and assessing the effect of medical cannabis and cannabinoids on pain. Of note, Wan et al. included all randomized clinical trials with more than 20 patients that compared medical cannabis or cannabinoids to any non-cannabis control for chronic pain at more than 1 month follow-up. The authors identified 32 studies, of which four specifically focused on cancer-related pain. The analysis of data from over 5,000 patients identified a small improvement of chronic pain as assessed by the visual analog scale compared to placebo (Wang et al., 2021). A recently published clinical study enrolled cancer patients undergoing treatment with medical cannabis and assessed multiple cancer-related symptoms at different timepoints. The study included 324 patients, of which 126 were available for all follow-ups. The authors concluded that most outcome measures improved significantly with medical cannabis treatment, with an 18% decline in symptom burden at the 6-months follow-up. Of note, the average weekly pain intensity was reduced with a median of 20% for approximately 80% of the study participants (Aviram et al., 2022).

Several preclinical studies have specifically focused on CIPN, a significant long-term issue in cancer survivors. Both AM1710, a selective CB-2 agonist and Δ9-THC were shown to be effective in treating paclitaxel-induced neuropathy, most likely via a CB2-linked mechanism (Deng et al., 2015). Intraperitoneal WIN 55212-2 significantly reduced thermal hyperalgesia and tactile allodynia in both sciatic nerve constriction and paclitaxel-induced neuropathy models with little to no side effects (Pascual et al., 2005). Vaporized cannabis plant (10.3% THC/0.05% CBD) was also effective in a similar setting, decreasing cold allodynia in paclitaxel-treated rats (Alkislar et al., 2021). Targeting the enzymes that degrade endocannabinoids has also been shown to be a valid approach for treating CIPN in several models of vincristine or cisplatin-induced allodynia (Masocha, 2018). Cannabinoids were even shown to have efficacy in preventing CIPN as reported by a 2014-study on paclitaxel-induced neuropathy where both WIN 55212-2 and AM1710 suppressed mechanical allodynia and cold allodynia at varying doses while the animals were receiving chemotherapy (Rahn et al., 2014).

In the clinical setting, there are some data pointing towards the efficacy of cannabinoids in treating CIPN. A small crossover pilot trial published in 2014 assessed nabiximols in cancer patients with CIPN and found that five of the 16 individuals enrolled experienced an average decrease of 2.6 points on a 11-point numeric rating scale for CIPN assessment (Lynch and Campbell, 2011). This was confirmed by a retrospective clinical analysis of cancer patients undergoing oxaliplatin-based chemotherapy. This retrospective study included over 500 patients and divided them based on their exposure to medicinal cannabis (prescribed for other conditions such as nausea or anorexia) in: a. cannabis exposure prior to oxaliplatin chemotherapy, b. cannabis exposure following the initiation of oxaliplatin treatment, and c. no exposure (control). The authors noted a decrease in neuropathy incidence in cannabis users, especially in those that had been exposed to cannabis prior to starting chemotherapy, suggesting a protective effect for cannabis in this setting (Waissengrin et al., 2021).

Topical cannabinoids have also been reported in several papers as being effective in CIPN. When applied to the tail, a solution of DMSO containing WIN 55212-2 was shown to significantly prolong response time in the tail-flick test, most likely through a CB1R-dependent mechanism (Yesilyurt et al., 2003). A case series published in 2021 included all cancer patients that reported the use of topical cannabinoids for alleviating CIPN in a US-based hospital between 2019 and 2020. Of the 26 patients included, 22 reported experiencing a benefit and 4 did not report any improvement in CIPN. Patients used a variety of cannabinoid-containing creams, mostly CBD or a mixture of CBD and THC and overall reported feeling an improvement minutes after topical administration with no side effects (D’Andre et al., 2021).

c) Opioid sparring effect—preclinical and clinical data

There is a plethora of preclinical studies exploring the effect of combining different cannabinoids with well-known analgesics, most often opioids (Schurman et al., 2020). For acute pain, Δ9-THC was shown to have a synergic effect with morphine in a rat model of arthritis (Cox et al., 2007) and in normal rodents undergoing the tail-flick test (Smith et al., 1998). The association between transdermal fentanyl or buprenorphine and Δ9-THC also yielded good results in hairless guinea pigs as assessed by the pin prick test. Intraperitoneal Δ9-THC (50 mg/kg) enhanced the potency of fentanyl 6.7-fold. More interestingly, transdermal Δ9-THC had even better results, with a 5.8-fold increase in potency for fentanyl and 7.2-fold increase for buprenorphine at 4 hours, suggesting significant benefits for a mixt opioid/cannabinoid analgesic patch (Cichewicz et al., 2005).

As such, there is abundant preclinical evidence for the opioid-sparing effect of over 20 natural and synthetic cannabinoids (Nielsen et al., 2022). Of note, this effect was reported irrespective of the type of opioid used. A large meta-analysis of available preclinical studies concluded that cannabinoids, in particular Δ9-THC could potentially have an opioid-sparing effect (Nielsen et al., 2017), thus prompting the design of several clinical trials.

One of the first randomized clinical trials to explore the potential synergic effect of opioids and cannabinoids was published in 2012. Adult cancer patients with moderate or severe cancer pain (an intensity of 4-8 on the numeric rating scale - NRS) despite adequate opioid treatment were randomized to receive an oro-mucosal spray containing either nabiximol (Sativex®) (low-dose, medium-dose or high-dose) or placebo for a period of 5 weeks. Over 250 patients were included in the study and results indicated a statistically significant analgesic effect of cannabinoids which was greatest in the low-dose group that received 1-4 sprays of 100 µL Nabiximols (1.6 points pain reduction on the NRS) (Portenoy et al., 2012). A larger clinical trial (phase three) with a similar design compared a self-titrated regimen of Nabiximols oromucosal spray (Sativex®) to placebo in opioid-treated cancer patients. Although cannabinoids were not found to be more effective than placebo in the statistical analysis of the primary efficacy endpoint, there was a numerical benefit of Nabiximols treatment, with an approximate decrease of 0.8–0.9 points on the NRS in the worst pain score (Lichtman et al., 2018). Two other phase three trials reported similar findings, with no clear-cut benefit for Nabiximols as a co-analgesic, especially in cancer patients requiring large doses of opioids (Fallon et al., 2018). However, a THC:CBD extract was shown to be effective in reducing pain when compared to placebo in cancer patients with intractable pain receiving opioids in a parallel group randomized study. A reduction of over 1.3 on the NRS was noted, with 43% of patients in the cannabis group reporting a reduction of more than 30% of their baseline pain score (Johnson et al., 2010). Despite this, a systematic review published by Hauser in 2019 concluded that when analyzing available data together for cancer patients receiving opioids, the addition of cannabinoids does not improve pain and does not reduce opioid usage (Häuser et al., 2019). More recently, the results of the MedCan1-CBD phase IIb study were published and the authors concluded that cannabidiol oil does not decrease opioid use in cancer patients (Hardy et al., 2023). Due to numerical improvement in NRS and a good safety profile, medical cannabis and cannabinoids have been classified in consensus statements in the “weak recommendation” category for patients with chronic cancer and non-cancer pain (Busse et al., 2021).

d) Approved medication

Although a number of countries authorized the use of Nabiximols (Sativex®) for multiple sclerosis-associated neuropathic pain, to date, there are no cannabinoid-based medications approved by FDA or EMA for the treatment of cancer pain.

As such, American Society of Clinical Oncology (ASCO), National Comprehensive Cancer Network (NCCN) and European Society for Medical Oncology (ESMO) have similar opinions for the clinical use of cannabinoids for this purpose. Clinicians should not offer cannabinoids for cancer pain, because it has no benefits, however also no harm (Loprinzi et al., 2020). Furthermore, there is need for further double-blind, placebo-controlled clinical trials with large sample size in order to establish proper indications (Fallon et al., 2018).

### 2.2 Chemotherapy induced nausea and vomiting

Chemotherapy-induced nausea and vomiting (CINV) is a significant issue for cancer patients, with 45%–65% of patients experiencing nausea and 15%–25% vomiting (Grassi et al., 2015). Although there are several effective antiemetic treatments and also several classes of drugs useful for preventing CINV, it remains an area of unmet need due to high costs, symptom under-reporting and challenges associated with treating the refractory forms of CINV (Gupta et al., 2021). Nausea is amongst the most feared side-effects of chemotherapy and can have a significant impact on patients’ quality of life (Sommariva et al., 2016). Aside from affecting regular activities, nausea and vomiting can also have serious somatic effects, such as dehydration, electrolyte imbalance, anorexia, esophageal tears, or fractures. It can also affect a patient’s willingness to continue a potentially curative anticancer treatment and, in time, it can lead to the deterioration of the patient’s physical and mental status (Lorusso et al., 2017).

The pathophysiology of vomiting involves a multistep process controlled by the cortex, triggered by afferent impulses to the vomiting center. Chemotherapy drugs trigger vomiting through several pathways, most often activating receptors in the trigger zone or stimulating intestinal vagal afferents (Navari and Aapro, 2016). The main neurotransmitters involved in the emetic response bind to serotonin, dopamine, acetylcholine, histamine, opioid and cannabinoid receptors (Gupta et al., 2021). There is a high concentration of endocannabinoids and CB-1R in both the chemoreceptor trigger zone and the dorsal vagal complexes (Behl et al., 2022), which has led researchers to investigate the anti-emetic properties of cannabinoids in the late 1970s, before the discovery of the 5-HT_3_ antagonists so broadly used nowadays. To date, CINV represents one of the therapeutic areas where the efficacy of cannabinoids has been clearly proven by both preclinical and clinical studies.

a) Preclinical and clinical studies

Reproducing CINV in an animal model is somewhat difficult, since mice and rats do not vomit in response to toxin exposure, thus requiring either the use of other species or evaluating other signs of chemotherapy-induced emesis. McCarthy and Borison used a feline model (cat) to compare the efficacy of cannabinoids (N-metyllevonantradol, nabilone) to prochlorperazine, the standard anti-emetic treatment in 1981. The authors concluded that cannabinoids have a significant dose-dependent effect on Cisplatin-induced vomiting which is superior to the effect of prochlorperazine (Colby et al., 1981). A dose of 0.05–1 mg/kg intraperitoneal THC was shown to inhibit CINV in a dose-dependent manner in a ferret model (Van Sickle et al., 2003), results that were confirmed in a Cisplatin-treated least shrew animal model (Ray et al., 2009). A 2004-study on house musk shrews compared Δ9-THC to ondansetron in CINV and found a comparable anti-emetic effect between the drugs. Interestingly, the authors also assessed the effect of THC/ondansetron combination and concluded that low, ineffective doses of these drugs become highly effective when administered together, thus suggesting synergy (Kwiatkowska et al., 2004). However, few years later, a somewhat similar study compared the anti-emetic efficacy of Δ9-THC, dexamethasone and tropisetron both individually and in combination in a Cisplatin-treated least shrew animal model (Wang et al., 2008). The authors reported that both Δ9-THC (59%–97% attenuation) and tropisetron (79%–100% attenuation) were effective individually, but their combination was not associated with synergic effects, results that do not concur with those of the former study, most likely due to different doses used.

Several synthetic cannabinoids with little or no psychotropic effect have also been assessed in CINV preclinical models with very good results. HU-210 was shown to be effective in alleviating both Cisplatin-induced and Emetine-induced symptoms in a pigeon CINV model (Ferrari et al., 1999). HU-211 was compared to Δ1-THC in a similar animal model and the authors concluded that pretreatment with 2.5 mg/kg of HU-211 decreased CINV by over 90%, whereas Δ1-THC failed to inhibit emesis in 50% of all animals tested and also had psychotropic effects (Feigenbaum et al., 1989). More recently, WIN 55212-2 was shown to prevent opioid-induced vomiting in a ferret model (SimoneauHamza et al., 2001).

One of the first clinical studies that showed the efficacy of cannabinoids dates back to the 90’s, when Gralla et al. (1999) compared THC to the anti-emetic standard of the time, which was high-dose metoclopramide. Results showed that THC was superior for CINV control in patients receiving Cisplatin. At the time, the body of research favoring the use of cannabinoids in CINV was very strong, which led to the approval of Dronabinol (synthetic THC) in this setting. Since its approval, there have been at least ten randomized, placebo-controlled trials assessing Dronabinol in cancer and non-cancer patients, most often with good outcomes and a satisfactory safety profile (Bajtel et al., 2022). Similarly, Nabilone was evaluated in several clinical trials and was found to be effective in the management of CINV, although most studies were somewhat underpowered (Cherkasova et al., 2022).

Several new cannabinoid-derived drugs have been tested in the clinical setting in recent years. Of those, Grimison et al. performed a phase II crossover study (81 participants) comparing THC and CBD mixture (capsules) to placebo in patients experiencing CINV during moderate-to-high emetogenic intravenous chemotherapy despite guideline-consistent antiemetic prophylaxis. The authors concluded that there is an important patient preference (85%) for THC-CBD capsules compared to placebo and a statistically significant improvement in nausea and vomiting for the experimental arm of the study (Grimison et al., 2020).

b) Approved medication

Dronabinol (Marinol® or Syndros®), was approved by the FDA in 1985 for treatment of nausea and vomiting associated with cancer chemotherapy, mostly after the failure of previous therapies (Warr and Hesketh, 2020). The Europeans are more restrictive with the use of Dronabinol, with some countries allowing its use only by special permit, or a temporary use authorization (Abuhasira et al., 2018).

Nabilone (Cesamet® or Canemes®), is approved for nausea associated with cancer chemotherapy. In comparison to Dronabinol, there are more European countries allowing the use of Nabilone in case of cancer patients (Abuhasira et al., 2018).

Oncology practice guidelines for clinicians, NCCN and ASCO include the indication of Nabilone and Dronabinol for nausea and vomiting refractory to conventional therapy. 2.5–5 mg oral solution Dronabinol should be used every 4–6 h 1–2 mg of Nabilone can be used twice daily as rescue therapy (May and Glode, 2016).

### 2.3 Anorexia and cachexia

Cancer-related cachexia and anorexia syndrome (CACS) is a complex wasting syndrome, commonly diagnosed in patients with advanced cancer, which leads to severe fat and muscle mass losses. The prevalence of cachexia in cancer patients increases from 50% to more than 80% as the disease progresses (Lim et al., 2020). Additionally, CACS is reported to be responsible for more than 20% of all cancer-related deaths (Onesti and Guttridge, 2014), thus representing one of the main challenges in oncology clinical practice.

Cachexia not only involves weight loss, but also anemia, electrolyte and water abnormalities, and is often accompanied by anorexia, defined as the loss of appetite and early satiety. Although anorexia is commonly associated with cachexia, the decrease in food intake is not the main culprit in CACS, as attempts to increase dietary intake through dietary counseling or nutritional supplementation are usually inefficient for stopping the wasting process (van de Worp et al., 2020). Chemotherapy can cause significant nausea and vomiting and subsequently lead to anorexia and weight loss. Additionally, oncology patients often experience psychological distress during their cancer journey, which is a known regulator of food intake.

Thus, CACS remains an extremely complex condition that has both objective and subjective features, encompassing a variety of alterations that range from physiological to behavioral and somatic distress (Arends et al., 2021). However, addressing CACS in all oncology patients is essential due to the important prognostic value and impact on the Quality of Life (QoL) of this condition (Kasvis et al., 2019). CACS management poses a significant challenge, as physicians have to address the low patient adherence and high drop-out rate of exercise and nutritional intervention strategies (Nishikawa et al., 2021), combined with the reduced efficacy and high incidence of side-effects of pharmacological treatments such as corticosteroids, progestins or antipsychotics (Arends et al., 2021). Moreover, because several mechanisms are involved, the likelihood of any one intervention to be successful is low (Argilés et al., 2019), opening the avenue for combined treatment approaches.

Historically, cannabis has been used as food, given it is an excellent source of fibers and oils. The oil from hempseed is very nutritious and contains a high quantity of omega-3-type fatty acids (Klumpers and Thacker, 2019), while hemp sprouts are rich in antioxidants (Cerino et al., 2021). Anecdotal evidence has long suggested that cannabinoids can increase appetite, with recreational cannabis users even reporting an enhancement of the sensory and hedonic properties of food (Kirkham, 2009). When studied in small trials and case series, THC appeared to improve appetite and attenuate weight loss. Studies suggest that cannabis could be used in anorexia and cachexia, mainly by stimulating appetite (due to cannabinoid receptors located in the CNS), but also by inhibiting the nausea mechanisms.

a) Preclinical and clinical data

Several animal models of CACS have been established, even though none can completely replicate all aspects of cancer cachexia that occurs in humans (Ng et al., 2023), most likely due to the syndrome’s complexity and the important emotional and psychological feature of CACS. Additionally, considering the large body of clinical data available, there are only few preclinical studies specifically designed for assessing the effect of cannabinoids on CACS. In an acute model of cachexia (rats that received 6 mg/kg intraperitoneal cisplatin), cannabigerol was shown to both increase food intake and prevent weight loss at 72 h, most likely by protecting against/reversing cisplatin-induced dysregulation (Brierley et al., 2019). A more recent translational study measured the serum levels of six CACS-related cytokines in colorectal cancer patients and in a CACS mouse model and found a negative correlation with body mass index. The authors also found that Δ9-THC treatment attenuated CACS-related muscle atrophy via an anti-inflammatory mechanism, together with decreasing the concentration of the afore-mentioned inflammatory cytokines, thus suggesting a CB2-mediated pathway of CACS (Ng et al., 2023).

Of note, preclinical data vary greatly in terms of anorexic/anti-anorexic effects of cannabinoids, most likely because the effect of cannabinoids on food intake is highly dependent on both dose and type of cannabinoid used. For example, 100 μg/kg of CP 55,940 (a synthetic cannabinoid) had significant anorexic effects and caused a decrease in body weight in rats (McGregor et al., 1996), whereas WIN 55212-2 (2 mg/kg) prevented the acceleration of gastric emptying induced by cachexia in rats (de Sousa Cavalcante et al., 2020). However, some of the candidates with positive results in the preclinical studies have translated towards clinical trials in both cancer and non-cancer patients.

Due to promising results in AIDS-related cachexia, Dronabinol has been assessed in several small clinical trials as an appetite stimulant in patients with CACS. A comprehensive review included all clinical data in the field and concluded that available evidence supports the use of Dronabinol in this setting (Voth and Schwartz, 1997). Subsequently, the North Central Cancer Treatment Group designed a large three-arm study to assess the effect of Dronabinol in cancer patients with CACS (Jatoi et al., 2002). The 469 patients enrolled were randomized to receive oral megestrol acetate, oral dronabinol or both and followed-up. The results, however, showed that megestrol acetate was superior to Dronabinol and that the combination of the two was not better than single drug megestrol therapy. Another phase III clinical study designed by the Cannabis-in-Cachexia-Study-Group compared oral cannabis extract, THC and placebo and found no significant difference between groups in terms of appetite or QoL (Cannabis-In-Cachexia-Study-GroupStrasser et al., 2006).

Despite these disappointing results, several other cannabinoids have been tested in clinical trials, some with positive outcomes. These significant variations most likely derive from differences in the active substance used, the dose and the administration route. There is no comprehensive data on the pharmacokinetic parameters of CBD in humans. T_max_ is shorter in smoking/inhalator and sublingual forms than in oral forms (1–6.12 h) (Lord et al., 2022). C_max_ is higher in the fed state than in the fasting state or in patients with liver dysfunction. Therefore, caution should be exercised in advanced cancer patients with low dietary intake (Millar et al., 2018). Another potential factor is the severity of cachexia, as more advanced or refractory cases are less likely to have benefits from any treatment. A retrospective analysis of cases from Santé Cannabis, a medical cannabis clinic in Canada, identified 54 individuals (43% of them had cancer) requiring cannabis for appetite improvement. Although there were no significant changes in weight between baseline and the 3-month assessment, patients reported a significant improvement in appetite, especially with nabilone and inhaled cannabis-based produce (Kasvis et al., 2019). In a more specific setting, Nabilone was compared to placebo in advanced lung cancer patients diagnosed with CACS and was shown to significantly increase caloric intake, double the carbohydrates intake, together with a significant increase in QoL (Turcott et al., 2018). A small pilot study enrolled advanced cancer patients with documented CACS and assessed the effect of cannabis capsules (95% THC and 5% CBD) on weight gain and several other patient-reported outcomes. Although the drop-off rate was quite high (11 out of the 24 patients that signed the consent form received the treatment for more than 2 weeks), the authors concluded that almost 20% of patients had a weight increase of more than 10% and almost 40% had stable weight at the time of study completion. Additionally, while receiving the capsules, cancer patients reported improvements in mood, pain and fatigue, as assessed by the EORTC QLQ-C30 questionnaire, albeit with some side effects (Bar-Sela et al., 2020).

There are a lot of uncertainties surrounding the potential effect of cannabinoids in CACS, as emphasized by a recently published systematic review and meta-analysis. After including all clinical trials with cannabinoids as appetite stimulants, the authors concluded that the level of evidence for different cannabinoids is either low or very low, underlining the need for additional studies and the high likelihood of recommendation changes if new data becomes available (Bilbao and Spanagel, 2022).

b) Approved medication

Dronabinol (Marinol® or Syndros®), is approved for anorexia associated with weight loss in patients with AIDS, but not for cancer patients. Current ASCO guidelines state that the administration of cannabinoids for anorexia/cachexia brings no benefits, given the fact that the strength of the evidence from the studies conducted until now is low (Roeland et al., 2020). Similarly, ESMO considers the existing literature data insufficient to justify medical cannabis or its derivatives use for treating anorexia or cachexia in cancer patients (Arends et al., 2021). However, the NCCN guidelines include the possibility of cannabinoid administration in the case of patients with a very low life expectancy (weeks, days until death) to stimulate the appetite.

### 2.4 Depression and anxiety

With early detection and new cancer therapies available, the life expectancy of cancer patients has increased, and many of them will live with cancer as a chronic disease controlled by ongoing therapy. Current estimates indicate that by 2040 approximately 26 million individuals will be living with and beyond cancer in the United States alone (Emery et al., 2022).

The prevalence of common mental disorders among people with cancer varies widely in the published literature. The mean prevalence of depression is believed to be around 13%, with literature data varying from 4% to 49% (Niedzwiedz et al., 2019).

Depression leads to a poorer QoL and compromises patient outcomes, with depression resulting in higher rates of mortality in cancer (Wang et al., 2020). A meta-analysis revealed that minor or major depression increases mortality rates by up to 39%, and that patients displaying even few depressive symptoms may be at a 25% increased risk of mortality (Satin et al., 2009). Additionally, depression triples the risk of nonadherence, further contributing to increased mortality (Fann et al., 2008).

Another important issue to consider is that depression often coexists with symptoms of anxiety in patients with cancer, with fewer available statistical data that examine anxiety alone. In case of cancer survivors, many of them experience cancer-related fear of recurrence, post-traumatic stress symptoms, anxiety, or depression after completing oncological treatment. In the study conducted by Götze et al. (2020) on 1,000 cancer survivors of mixed tumor entities 10 years after the initial diagnosis, results showed a prevalence as high as 17% and 9% for depression and anxiety, respectively. Similar to the other conditions described previously, cannabis has a long history of use as an anxiolytic and antidepressant remedy (Zuardi, 2006), (Russo and Pertwee, 2014).

a) Preclinical and clinical data

Recent experimental data seem to confirm the physiological substrate of these effects. The endocannabinoid system is heavily involved in mood regulation and high levels of CB receptors have been described in the limbic system and prefrontal cortical areas (Maldonado et al., 2020). In a preclinical model for assessing anxiety, low doses of THC (0.3 mg/kg) were associated with anxiolytic-like responses in male CD1 mice (Berrendero and Maldonado, 2002). Similar results were observed after intraperitoneal administration of varying THC doses (0.75 mg/kg being the most effective) in rats, most likely linked to CB1R activation (Rubino et al., 2007).

Additionally, antidepressant effects of cannabinoid administration have been reported in preclinical studies. WIN 55212-2 significantly improved results in the rat forced-swim test (a murine model of depression) when administered intraperitoneally 0.75, 5 and 23 h before the test (Bambico et al., 2007). The preclinical data that supports potential benefits of CBD on both depression and anxiety is perhaps even more convincing, as highlighted in a comprehensive review performed by de Mello Schier et al. (2014).

Of note, the effect of cannabinoids on anxiety and depression seems to be dose-dependent and cannabinoid-dependent, similar to results reviewed for other symptoms. For example, a large dose of the synthetic cannabinoid CP 55940 (75 μg/kg) induced anxiogenic effects in male rats; however, the 10 μg/kg dose did not yield the same effect (Marco et al., 2003) and a dose of 1 μg/kg was associated with behavior suggestive for anxiolysis (Marco et al., 2004).

The dose- and strain-dependent anxiogenic or anxiolytic effect of cannabinoids are similar to data from real-world reports of recreational cannabis use, where some individuals report feeling relaxed, whereas others experience paranoia, panic and anxiety (Freeman et al., 2015), most likely depending on both individual variations and specific CBD/THC content of the drug. Although there are several large on-going randomized clinical trials assessing the effectiveness of different cannabinoids on depression and anxiety, currently available clinical data are sparse and heterogenous. Nonetheless, most authors concur that CBD does indeed have anxiolytic properties and can even reverse some of the negative psychological effects of smoking marijuana. A study on over 100 cannabis users assessed both memory and psychotomimetic symptoms in acutely intoxicated and drug-free states. The authors also analyzed the content of both CBD and THC within the smoked cannabis. Results showed that while there were no significant differences in terms of THC, CBD concentrations varied significantly within different types of cannabis. The low-CBD group had significantly higher ratings of anxiety compared to the high-CBD group and higher levels of CBD seemed to be protective against cannabis-induced memory impairment (Morgan et al., 2010). A more recent study, however, assessed distress-related symptoms in patients using inhaled cannabis and reported that over 95% of the 670 individuals enrolled experienced a statistically significant decrease in symptom intensity, particularly for agitation and anxiety, results that correlated with THC and not CBD concentrations (Stith et al., 2020). A recent comprehensive review identified ten clinical studies that assess the relationship between cannabis and anxiety and concluded that cannabinoid-based treatment, especially that which contains CBD, could represent a therapeutic option for people with pre-existing anxiety (Sharpe et al., 2020).

The effect of cannabinoids on depression is less clear in the clinical setting, as increased cannabis use and depression seem to co-occur quite often (Feingold and Weinstein, 2021). Additionally, there are a lot of hindrances for this area of research, especially due to concurrent anti-depressant medication, patient adherence and various cannabinoids/preparations/plant strains being used, which has led to contradictory results. For example, a small study on 40 multiple sclerosis patients with depression and chronic cannabis use concluded that withdrawal from cannabis improved depression scores at the 4-week landmark assessment (Feinstein et al., 2021). On the other side, a recently published observational trial of cannabis users versus controls concluded that medicinal cannabis users had lower depression scores. Moreover, the individuals in the control group that initiated treatment with medicinal cannabis whilst on the study reported decreased depression and anxiety scores (Martin et al., 2021).

b) Approved medication

Currently, no cannabinoid is approved for managing anxiety or depression outside a clinical trial, with no information regarding the use of cannabinoids for this indication in the previously mentioned guidelines. However, a recently published paper presented the Multinational Association of Supportive Care in Cancer (MASCC) guidelines for cannabis and insomnia, anxiety, or depression. The authors concluded that currently it is impossible to have a guideline about the use of cannabinoids in the treatment of depression and anxiety in patients with cancer due to insufficient data. However, they recommend that patients should not be routinely advised to stop cannabis if already on cannabis and are experiencing benefits with no bothersome adverse effects (De Feo et al., 2023).

## 3 Cannabinoids as anti-cancer drugs

Cancer-attributed medical care costs in the United States are substantial and projected to increase dramatically by 2030 to 246$ billion, mainly due to population growth, reflecting the rising burden of cancer care among cancer survivors (Mariotto et al., 2020). As a result, scientists are trying to identify new therapies with maximum antitumor effect and with the least adverse effects, to ensure the QoL of these patients.

Among chat groups, social media or documentaries for cancer patients, a lot of information is shared according to which cannabis can cure cancer. Indeed, the literature provides a consistent trail of *in vitro* and *in vivo* studies in which cannabinoids can alter tumor dynamics. Preclinical data shows that through receptors located on the surface of tumor cells, cannabinoids modulate a series of intracellular signaling pathways that trigger a wide range of anti-oncogenic effects. These include inducing programmed cell death by apoptosis, blocking cell proliferation, decreasing tumor angiogenesis and inhibiting cancer cell migration, invasiveness and metastasis. Ultimately, these actions can indeed lead to a reduction of tumor growth, observed in both *in vitro* and *in vivo* studies (Velasco et al., 2012).

However, it is known that promising preclinical results do not always readily or easily translate into real world clinical benefit. Rising prevalence and demand for cannabis in recent years has driven some patients to shun conventional cancer treatments in favor of different cannabinoid preparations, in hopes of curing their disease. Given the insufficient scientifically validated clinical data, the empirical administration of cannabinoids, to the detriment of conventional therapy, is truly worrisome. When discussing treatment possibilities with patients, clinicians should have enough information in order to correctly and completely inform the patient about the real benefits and potential risks of using cannabinoids together with or instead of approved anti-cancer treatments.

### 3.1 The endocannabinoid system: mechanisms and signaling pathways

The relationship between the endocannabinoid system (ECS) and carcinogenesis, tumor growth and metastasis is extremely complex and not fully understood, especially since the definition of the ECS is constantly expanding to include enzymes involved in endocannabinoid synthesis and degradation, endocannabinoid-like lipid mediators and non-CB receptors that bind to cannabinoids, such as the TRPV channel subfamily or the G protein-coupled receptors (GCPR) (Filipiuc et al., 2021).

Both CB receptors and endogenous cannabinoid levels are altered in several cancer cell lines. An increase in CB2R expression in the tumor has been linked to increased aggressiveness and recurrence risk in breast, prostate, pancreas, thyroid and colon cancer, whereas the relationship between CB1 expression and tumor progression is less clear (Pagano et al., 2021).

A large body of available data shows that cannabinoids can reduce cancer cell proliferation through protein kinase B (Akt) inhibition (Hinz and Ramer, 2022). Akt is a serine/threonine kinase that plays a key role in growth factor-induced cell survival. It is a critical mediator of the canonical PI3K signaling pathway, which has long been recognized for its major oncogenic role within the cell (Revathidevi and Munirajan, 2019). Other important mechanisms include retinoblastoma protein hypophosphorylation (Hinz and Ramer, 2022), promotion of reactive oxygen species (Dando et al., 2013), modulation of the mitogen-activated protein kinases (MAPK) pathway and apoptosis signaling. Several preclinical studies report that cannabinoids induce cell cycle arrest by downregulation or inactivation of cyclin-dependent kinases (CDK) and cyclin modulation (Thoma et al., 2021). Depending on the cancer cell line, the type of drug and the dose used, available data indicate that cannabinoids can downregulate CDK2 (Caffarel et al., 2006), which in turn induces cell cycle arrest and affects proliferation. Another relevant mechanism in ECS signaling is ceramide synthesis. Ceramide is a sphingolipid that acts as a second messenger in activating the apoptotic cascade, thus playing a key role in programmed cell death (Mullen and Obeid, 2012). Several cannabinoids and endocannabinoids can elicit ceramide production from membrane phospholipids, which determines cell autophagy (Brown et al., 2013).

Cannabinoids can also modulate cancer progression via the increase of intercellular adhesion molecule-1 (ICAM-1) expression, decreasing metalloproteinase expression and modulating several other extracellular matrix components (Ramer et al., 2012). The ECS system has also been shown to downregulate a number of angiogenesis-inducing factors such as angiopoietin-2, placental growth factor and vascular endothelial growth factor (VEGF) that play a key role in tumor growth and new vessel formation (Hinz and Ramer, 2022).

However, more research is needed to define the ECS’ role in tumor heterogeneity and how its signaling impacts the activation of other signaling pathways involved in cancer dynamics. How cannabinoids are involved in host metabolism via their GPCR receptors, as they present a role in activating numerous receptor tyrosine kinases and Toll-like receptors in the induction of altered epigenetic landscape in cancer cells, represents an exciting area for investigation, as it could modify cancer metabolism and epigenetic reprogramming to a metastatic phenotype. This means that in the future, focus on epigenetics could be an innovative target for preventing and treating cancer.

While most reports indicate an anti-tumor effect, others have also shown that in certain cell lines and in certain concentrations, some cannabinoids can also have the opposite effect, promoting cancer progression via MAPK pathway (Liu et al., 2020). Moreover, over-activation of the ECS has been demonstrated in several tumor types, thus further emphasizing the potential pro-tumorigenic effect of cannabinoids. Last, but not least, the complex relationship between the ECS and the immune system is another potential hindrance for the clinical use of cannabinoids as anti-cancer drugs, since they might induce immunosuppression (Moreno et al., 2019).

However, more specific mechanisms of the endocannabinoid system on host metabolism and energy homeostasis remain to be elucidated. In addition to agonists, antagonists, and inverse agonists as targets for cannabinoids in oncology, allosteric modulators have been identified. They have been shown to bind topographically distinct sites from the classical orthosteric sites, and as in consequence, can contribute to the tempering of the cannabinoid receptor signaling without the desensitization, tolerance and dependence (Shore et al., 2014). Allosteric modulators have numerous advantages, as they act only on tissues where ECs (lipid mediators, e.g., anandamide (AEA) and 2-arachidonoylglycerol (2-AG)) are present. Especially in cancer, where these components of the endocannabinoid system can vary between so many factors and consequently between each patient, the use of allosteric modulators is even more advantageous, as the entity and the duration of their effect will depend on the ECs tone at that time and selective to that area, consequently minimizing the off target side-effects (Burford et al., 2015). Currently some CB1R allosteric modulators have been discovered and studied, while very few compounds have been identified as CB2R allosteric modulators (Gado et al., 2019).

### 3.2 *In vitro*/*in vivo*/clinical data supporting the antitumor activity of cannabinoids

Emerging data regarding the effect of cannabinoids on key cancer signaling pathways has generated significant research interest over the past decades. While a lot of data comes from glioma cell lines, several other tumors have since been assessed via *in vitro* models. Table 1 summarizes the current state of preclinical research on antitumor effects.

**TABLE 1:** Current state of the preclinical research about the antitumoral effects of cannabinoids, both *in vitro* and *in vivo*.

	Induction of autophagy and apoptosis	Reduction of inflammation and inhibition of proliferation	Inhibition of angiogenesis, tumor invasiveness, and metastasis	Interactions with the immune system
Breast cancer	*In vitro*	*In vitro*	*In vitro*	*In vitro*
	• Induced apoptosis and cell cycle arrest at G2/M phase Caffarel et al. (2006)	• Inhibited cell growth and proliferation Ligresti et al. (2006), Takeda et al. (2014)	• Inhibited proliferation, migration and invasion McAllister et al. (2007), Elbaz et al. (2015), García-Morales et al. (2020)	• Inhibition of antitumor immune response via enhancement of Th2-associated cytokines McKallip et al. (2005)
	• Induced apoptosis and autophagy (Ligresti et al. (2006), Hernán Pérez de la Ossa et al. (2013b)	• Inhibited estradiol-induced proliferation Takeda et al. (2014), von Bueren et al. (2008)	• Diminished cancer cells chemotaxis Coke et al. (2016)	
		• Increased proliferation and tumor growth McKallip et al. (2005), Takeda et al. (2009)	• Inhibition of tumour induced angiogenesis Picardi et al. (2014)	
	*In vivo*	*In vivo*	*In vivo*	
	• Reduced breast cancer progression through Akt inhibition and apoptosis Caffarel et al. (2010)	• Reduced tumor growth Caffarel et al. (2010), Blasco-Benito et al. (2018), Blasco-Benito et al. (2019), Hirao-Suzuki et al. (2019)	• Increased tumor growth and metastasis McKallip et al. (2005)	
			• Inhibited tumor angiogenesis Caffarel et al. (2010)	
			• Inhibited tumor growth, migration, invasion, and	
			• metastasis Elbaz et al. (2015)	
			• Increased survival and decreased metastasis Murase et al. (2014)	
Brain cancer	*In vitro*	*In vitro*	*In vitro*	*In vitro*
	• Induced apoptosis Sánchez et al. (1998), Galve-Roperh et al. (2000), Carracedo et al. (2006a), Hernández-Tiedra et al. (2016), Meyer et al. (2018)	• Inhibited cell viability and proliferation, dose-dependent Jacobsson et al. (2000), Goncharov et al. (2005), McAllister et al. (2005), Marcu et al. (2010)	• Reduced invasion Fisher et al. (2016), Alharris et al. (2019)	• Angiogenesis and glioma cells growth via microglial M2 polarization (Lei et al. (2020)
	• Induced autophagy via ceramide accumulation and ER stress (Carracedo et al. (2006a), Salazar et al. (2009)			
	• Inhibited cell proliferation and induced apoptosis (Massi et al., 2004; Marcu et al., 2010; Nabissi et al., 2013; Scott et al., 2014; Fisher et al., 2016; Alharris et al., 2019)			
	• Induced autophagy Ellert-Miklaszewska et al. (2021), Huang et al. (2021), Nabissi et al. (2015), Ivanov et al. (2021)			
	*In vivo*	*In vivo*	*In vivo*	*In vivo*
	• Upregulated stress protein p8 and induced apoptosis Carracedo et al. (2006a)	• Reduced tumor growth Massi et al. (2004), Salazar et al. (2009), Hernán Pérez de la Ossa et al. (2013a)	• THC-loaded nanoparticles reduced cell proliferation, angiogenesis, and increased apoptosis Hernán Pérez de la Ossa et al. (2013a)	• Angiogenesis and glioma growth via microglial M2 polarization Lei et al. (2020)
	• Induced autophagy Nabissi et al. (2015), Hernández-Tiedra et al. (2016)	• Reduced tumor growth Hernán Pérez de la Ossa et al. (2013a), Massi et al. (2004)	• Inhibition of the VEGF factor pathway Blázquez et al. (2004)	
	• Enhanced apoptosis and decreased a ngiogenesis McAllister et al. (2005), Hernán Pérez de la Ossa et al. (2013a)			
Lung cancer	*In vitro*	*In vitro*	*In vitro*	*In vitro*
	• THC-loaded nanoparticles exhibited significant cytotoxicity Baram et al. (2019)	• Low levels induced cell proliferation or did not decrease cell survival Hart et al. (2004), Baram et al. (2019)	• Inhibited cell proliferation, chemotaxis and invasion (Preet et al. (2008), Milian et al. (2020)	• Increased lymphokine-activated killer cells via upregulation of ICAM-1 Haustein et al. (2014)
	• Induced apoptosis Ramer et al. (2013)	• Increased susceptibility to lysis by lymphokine-activated killer cells Haustein et al. (2014)	• Reduced migration Milian et al. (2020)	• Tumor growth acceleration based on reduced tumor immunogenicity Zhu et al. (2000)
		• Inhibits cell proliferation through Akt/PI3K and JNK pathways BoyacıoğluBilgiç et al. (2021)	• Reduced invasion, metastasis, migration, and restored epithelial phenotype McMahon et al. (2001), Ramer and Hinz (2008), Ramer et al. (2010b), Milian et al. (2020)	
	*In vivo*	*In vivo*	*In vivo*	
	• THC-loaded nanoparticles exhibited significant cytotoxicity Martín-Banderas et al. (2015)	• Increased tumor growth and reduced tumor immunogenicity Zhu et al. (2000)	• Inhibited tumor growth and metastases Preet et al. (2008)	
		• Decreased tumor growth Ramer et al. (2010a), Ramer et al. (2013)	• Decreased metastasis Ramer and Hinz (2008)	
Digestive cancers	*In vitro*	*In vitro*	*In vitro*	*In vitro*
	• Decreased cell viability and induced autophagy for hepatocellular cancer Vara et al. (2011)	• THC-loaded microspheres inhibited proliferation (Hernán Pérez de la Ossa et al. (2013b)	• Reduced proliferation, migration, invasion, and induced apoptosis for hepatocellular cancer (Leelawat et al., 2010)	• Reduced expression of PDL-1, thereby enhancing immune checkpoint blockade of pancreatic cancer cells (Yang et al., 2020)
	• Decreased cell viability in pancreas cancer (Carracedo et al., 2006b)	• Inhibits proliferation through Akt pathway in hepatocarcinoma (Rao et al., 2019)		
	• Reduced cell proliferation, promoted apoptosis and elevated ROS levels in colon cancer (Aviello et al., 2012; Honarmand et al., 2018)	• Cell cicle arrest in gastric cancer (Zhang et al., 2019)		
	*In vivo*	*In vivo*	*In vivo*	*In vivo*
	• AMPK- dependent activation of autophagy in hepatocellular carcinoma (Vara et al., 2011)	• Reduced hepatocellular tumor growth (Vara et al., 2011)	• Decreased metastasis and angiogenesis in colon cancer (Honarmand et al., 2018)	• Regulation of tumor-immune microenvironment in pancreatic cancer (Qiu et al., 2019)
		• Reduced the growth of tpancreativ tumors (Carracedo et al., 2006b)		
		• Reduced aberrant crypt foci polyps and tumor growth (Aviello et al., 2012; De Petrocellis et al., 2013; Honarmand et al., 2018; Jeong et al., 2019)		
Gynecological and urogenital cancers	*In vitro*	*In vitro*	*In vitro*	
	• Inhibited cell growth and induced apoptosis in endometrial (Fonseca et al., 2018) and cervical cancer cells (Lukhele and Motadi, 2016)	• Cell cycle arrest in prostate cancer (Roberto et al., 2019)	• Reduced invasion via increased TIMP-1 expression in cervical cancer cells (Ramer and Hinz, 2008)	
	• Decreased cell viability in prostate cancer cells (De Petrocellis et al., 2013; Baram et al., 2019)		• Impairment of invasive capacity of prostate cancer cells (Pietrovito et al., 2020)	
	• Induced apoptosis in prostate cancer cells (Ruiz et al., 1999)		• Cell migration inhibition of urothelial cell carcinoma (Anis et al., 2021)	
			• Diminished cancer cells chemotaxis in prostate cancer cells (Coke et al., 2016)	
	*In vivo*	*In vivo*	*In vivo*	
	• *N/A*	• N/A	• *N/A*	

Munson et al. (1975) were among the first researchers, who in 1975 observed the antitumor effects of cannabinoids. They administered Δ9-THC, Δ8-THC and cannabinol to Lewis lung adenocarcinoma and observed a decrease in cell proliferation.

Since then, more studies documented the antiproliferative effects of cannabinoids. Kolbe et al. (2021) recently presented the effects of Δ9-THC in glioblastoma multiforme (GBM) and the ability to modify the number of Ki67^+^ cells of human patient-derived GBM cells, particularly through the activation of the orphan receptor GPR55 without affecting CB1R and CB2R as usual targets. Their findings suggest that the sensitivity of cannabinoids and receptor-dependent signaling pathways should be considered to reflect the heterogeneity amongst GBM forms which is critical for when evaluating this translationally to clinic.

Synthetic cannabinoid WIN 55212–2 was also used in case of patient-derived glioblastoma multiforme cells, and was associated with the induction of autophagy and apoptotic cell death, at doses between 0.1 nM and 2 μM (Ellert-Miklaszewska et al., 2021).

Cell death via proapoptotic and anti-proliferative activity of 24 cannabis extracts on head and neck squamous cell carcinoma, had an effect proportional to the CBD content. Extract with the highest CBD content possessed the highest cytotoxic effect, while extracts with minimal CBD had a neglectable cytotoxic effect (Blal et al., 2023).

In case of breast cancer, doses as low as 1–5 µM of CBD induced significant cell death (Kosgodage et al., 2018). CBD’s IC50 values for most cell lines are consistently low, indicating that breast cancer cell lines are generally sensitive to CBD’s anti-proliferative effects (Shrivastava et al., 2011). CBD induced inhibition of cell proliferation and invasion on a human breast adenocarcinoma cell line, with inhibitory concentrations of only 1.5 μM (Nallathambi et al., 2018). In hepatocellular carcinoma cell lines, treatment with Δ9-THC and JWH-015 reduced cell viability through activation of the CB2R (Vara et al., 2011).

In addition to the *in vitro* testing, researchers explored the possibility that cannabinoids could influence the tumor dynamics, in *vivo* studies. The ability of THC to treat ErbB2-positive breast cancer, has been evaluated in some recent studies. For example, in a mouse model of ErbB2-driven metastatic breast cancer, THC treatment was able to reduce tumor growth, as well as the amount and severity of lung metastases. THC treatment also induced apoptosis and limited tumor angiogenesis (Caffarel et al., 2010). *In vivo* mouse models of lung cancer showed that treatment with 10 mg/kg/day of CBD resulted in reduced cell viability, decreased overall tumor growth and decreased metastasis (Ramer et al., 2010a). Similarly, murine models of glioblastoma multiforme revealed that treatment with CBD was able to inhibit tumor growth, enhance apoptosis and significantly prolong mouse survival (Hernán Pérez de la Ossa et al., 2013a; Singer et al., 2015).

Even though recent results in preclinical studies are increasing in number, the existing clinical trials investigating the anti-cancer effects of cannabinoids as single agents are very few and all so far have failed to show any high-quality positive effects (Table 2). However, the study conducted by Guggisberg&collab, includes data from 77 peer-reviewed case reports studies, and concluded that 81% of cases lacked sufficient evidence to support claims that cannabis had an anticancer effect (Guggisberg et al., 2022).

**TABLE 2:** Clinical trials (finished or ongoing) for potential use of cannabinoids for their antitumor effects.

Cancer type	Type of study	Intervention/Treatment	Outcome measure	Result	Citation/Clinical trial #
Glioblastoma multiforme	*Part 1*- Phase 1b (open label)	Nabiximols oromucosal spray in combination with dose-dense Temozolamide	• Adverse events	• In *Part 1* most adverse events were mild (CTCAE grade 1) or moderate (grade 2), the most frequently reported being fatigue, dizziness, headache, and vomiting	Twelves et al. (2021)
	*Part 2 *randomized, double-blind, and placebo-controlled		• Progression Free Survival at 6 months (PFS)	• In *Part 2*, 33% of both nabiximols- and placebo-treated patients were progression-free at 6 months. Survival at 1 year was 83% for nabiximols- and 44% for placebo-treated patients	
			• Overall Survival (OS)		
Glioblastoma multiforme	Phase II, multi-center, double-blind, placebo-controlled, randomized trial (ARISTOCRAT)	Sativex in combination with Temozolamide	• Overall survival time (OS)	• Ongoing	NCT05629702
			• Progression-free survival time (PFS)		
			• Health-related quality of life (HRQoL)		
			• Adverse events		
Glioblastoma multiforme	Phase Ib, Open-label, Multicenter	THC+CBD 1:1 combination in the presence of temozolomide and radiotherapy	• Adverse events	• Ongoing	NCT03529448
			• THC-CBD Maximum tolerated dose		
			• Tumor volume		
			• Overall survival		
			• Progression free survival		
Brain cancer	Phase 1 open label	Dexanabinol	• Dose Limiting Toxicity	• Generally, well tolerated	Juarez et al. (2021)
			• Pharmacokinetics parameters of Dexanabinol		
			• Adverse Events	• No objective tumor responses occurred	
			• Progression Free Survival		
Glioblastoma multiforme	Phase II double-blind randomized clinical trial, single-centre	Whole plant extracts of cannabis based on a 1:1 and 4:1 ratio of THC: CBD in combination with standard of care	• Side effects	• Both cannabis product ratios were found to be well tolerated	Schloss et al. (2021)
			• Blood safety markers		
			• Dose response	• 11% had a reduction in disease, 34% had stable disease, 16% had slight tumor enhancement, 10% had progressive disease	
			• Tumour growth		
Glioblastoma multiforme	Pilot phase I trial	Delta-9-THC	• Safety of intracranial THC administration	• No psychoactive side effects	Guzmán et al. (2006)
			• Overall Survival	• No clinical benefit	
Solid tumors	Phase 2 open label	Cannabidiol	• Overall Response Rate	• Unknown	NCT02255292
Glioblastoma Multiforme, Multiple Myeloma, and GI Malignancies	A Randomized, Double-Blind, Placebo-Controlled, Parallel, Multi-Cente	Cannabidiol (BRCX014) in combination with standard of care treatment	• Overall response rate	• Unknown	NCT03607643
			• Time to progression (TTP)		
			• Progression-free survival (PFS)		
			• Quality-of-life		
Acute leukemia and myelodysplastic syndrome	Phase 1, Phase 2 open label	Cannabidiol with standard GVHD prophylaxis (cyclosporine and methotrexate)	• Incidence rate of acute graft-versus-host disease after allogenic hematopoietic stem cell transplantation (GVHD)	• Cannabidiol prevents GVHD	Yeshurun et al. (2015)

Different combinations between cannabinoids and anti-cancer drugs are being explored based on cannabinoid’s potential for increasing the sensitivity of cancer cells to therapy, thus significantly increasing drug efficacy. There are several small clinical trials/case series available in the literature that suggest a potential role for this combined approach. For example, CBD/gemcitabine combination is more effective than single-drug gemcitabine, most likely because CBD can modulate ERK activation, a common mechanism to acquire chemoresistance both *in vitro* and *in vivo* (Adamska et al., 2018; Domenichini et al., 2019).

Co-administration of Doxorubicin and CBD in hepatocarcinoma (known for its chemoresistance) was shown to enable the internalization of Doxorubicin into the tumor cells, thus enhancing its action. This facilitated uptake might allow the use of lower Doxorubicin doses and therefore may improve the therapeutic index, decreasing chemotherapy-associated resistance (Neumann-Raizel et al., 2019). A similar result was reported for glioblastoma cell lines. Nabissi et al. (2013) provided evidence that addition of CBD increased the susceptibility of human glioblastoma cells to chemotherapeutic agents carmustine (BCNU), doxorubicin (DOXO) and temozolomide (TMZ).

In case of myeloma cell line, Bardabo et al. showed that synthetic cannabinoid WIN 55212–2 increased the sensitivity of the cells that were resistant to melphalan or dexamethasone, consequently increasing the anti-cancer activity of the chemotherapy (Barbado et al., 2017).

In a glioma xenograft model, Torres et al. (2011) demonstrated that the combination of THC with temozolamide (TMZ) inhibited tumor growth. Furthermore, combined treatment with these two agents exhibited much higher growth inhibition that each of them alone. In line with this study, Valero et al. showed that oral administration of Sativex, enhanced the effect of TMZ in glioma xenograft models (López-Valero et al., 2018).

Evidence from *in vitro* and *in vivo* studies can indicate which cannabinoid combination has the strongest possibility for successful translation to clinic. However, controlled clinical trials are required to test the value of these combinations in the cancer setting. There are several clinical trials which evaluate the antitumor capacity of different cannabinoids, when paired with standard treatment.

Table 2 summarizes some of the completed or ongoing clinical trials regarding the potential use of cannabinoids for their antitumor effects.

Of these, most are centered around glioblastoma patients due to good preclinical and clinical data already available. A recently published randomized, double-blind, and placebo-controlled phase 1b clinical study investigated the safety and preliminary efficacy of nabiximols oromucosal cannabinoid spray and dose intense (DIT) TMZ in patients with first recurrence glioblastoma. Nine of the 12 nabiximols and 6 of the 9 placebo recipients progressed by 6 months. Despite the similar progression rates, 1-year survival was 83% for the nabiximols patients and 44% for the placebo group (*p* = .042). Moreover, the trend persisted so that at 2 years, 50% of the nabiximols recipients were still alive compared to 22% of the placebo group (*p* = .134) (Twelves et al., 2021).

An ongoing clinical study, with 21 patients enrolled, investigates the antitumor activity of orally administered Sativex, in addition to dose-intense Temozolamide in recurrent glioblastoma patients. This open-label study also assesses the frequency and severity of adverse events in patients receiving Sativex in combination with standard chemotherapy compared to placebo-chemotherapy (NCT01812616). ARISTOCRAT is a phase II, multi-center, double-blind, placebo-controlled, randomized trial that aims to compare Sativex with placebo in patients with recurrent MGMT methylated glioblastoma treated with temozolomide (TMZ). The trial will randomize a target number of 234 patients on a 2:1 basis to receive either Sativex or Sativex-matched placebo, in combination with standard TMZ (NCT05629702). Another ongoing phase Ib, open-label, multicenter, intrapatient dose-escalation clinical trial investigates the safety profile of the THC+CBD combination at a 1:1 ratio, adding temozolomide and radiotherapy in patients with newly diagnosed glioblastoma (NCT01812603).

As previously mentioned, several key elements of the endocannabinoid system are expressed throughout the immune system and cannabinoids play an important role in immunomodulation. High concentrations of THC suppress the activity of T and B cells, while low concentrations have been associated with immunostimulatory effects (Pryimak et al., 2021). The possibility of using cannabinoids as neoadjuvant therapy together with biologics represents a fruitful avenue for future research (Pryimak et al., 2021). It has been shown that immunotherapy alters serum concentrations of endogenous CB independently of cannabinoid administration (Bar-Sela et al., 2020). However, the scarce clinical data available more likely indicate a deleterious effect of cannabinoids on tumors exposed to immunotherapy. A study by Taha et al. concluded that concomitant administration of cannabinoids and nivolumab immunotherapy reduces response rates (Taha et al., 2019). Other reports underlining the poor clinical outcome and reduced survival in cases where checkpoint inhibitors and cannabinoids were combined have been published (Bar-Sela et al., 2020; Xiong et al., 2022) Some data suggest that THC stimulates breast cancer growth by reducing antineoplastic immune responses (Hinz and Ramer, 2022).

Regarding the potential application of cannabinoids in combination with radiation therapy, even fewer studies have been published. Scott et al. investigated whether cannabinoids could enhance the cytotoxic effects of irradiation. Results were observed *in vivo*, where the triple combination of CBD, THC and irradiation significantly reduced the tumor size in an orthotopic syngeneic glioma mouse model, with the schedule of administration being of utmost importance (Scott et al., 2014).

## 4 The risk of tumor growth or development of a synchronous cancer—preclinical and clinical data

Despite an ever-increasing body of evidence that cannabinoids may have anti-tumor activity, it seems that this effect is highly dependent on several factors. One possible explanation for this variation is the high heterogeneity of the ECS system and its change in relationship to cancer progression. Additional factors to consider are the lack of consensus on anti-cancer cannabinoid responses, different drugs and doses used or even the experimental variability among scientists.

Cannabinoids have been shown to produce a biphasic effect depending on the concentration of the compound used. This translates into a delicate balance between the pro- and antitumor effects, with high concentrations (micro-molar) of endogenous cannabinoids displaying an inhibitory effect on tumor growth, whereas low concentrations (nano-molar) induce cell proliferation (Katsidoni et al., 2013).

In case of a triple negative breast cancer cell line, McAllister et al. (2007) demonstrated that higher concentrations of CBD caused a significantly higher decrease in cell proliferation and invasion as opposed to lower concentrations. Another study evaluating the effect of CBD on the tumor microenvironment revealed that lower concentrations of CBD had less of a direct effect on proliferation in comparison to higher concentrations (Elbaz et al., 2015). Further, Hart et al. (2004) showed that treatment of cancer cells with THC, HU-210, WIN 55212-2 and AEA stimulated mitogenic signaling.

On the other hand, the antitumor effects of cannabinoids may vary not only based on the type of cannabinoid, but also depending on certain characteristics of the targeted cells. The study conducted by Baram et al. (2019) assessed the antitumor effects of 12 whole cannabis extracts containing significant amounts of different phytocannabinoids on 12 different cancer lines from various tumor origins. They found a heterogenous antitumoral response between the Cannabis extracts and also between the cancer cell lines derived from the same organ.

There is also data mentioning that smoking cannabis could increase the risk of developing cancer. Two proto-oncogenes are overexpressed in the bronchial epithelium of cannabis-only smokers when compared with tobacco-only smokers (Barsky et al., 1998). The net association between cannabis use and developing cancer is, however, unclear. Cannabis and tobacco smoke share carcinogens, including toxic gases, reactive oxygen species, and polycyclic aromatic hydrocarbons (Hoffmann et al., 1975), believed to be 20 times higher in unfiltered marijuana than in cigarette smoke (Moir et al., 2008). Maertens et al. (2013) found that both cannabis and tobacco smoke influence the same molecular processes, but have differences in terms of activation of pathways, which could explain the contradicting information in the literature.

Studies of large databases have also yielded different results. 64,855 members of the Kaiser Permanente Medical Care Program, former or current cannabis users, were included in a study on the incidence of cancer. After an 8.6 years follow up, it was concluded that there was no increased risk of respiratory cancer (Sidney et al., 1997). By contrast, Zhang et al. (1999) found a correlation between cannabis users and squamous cell carcinoma of the head and neck.

The systematic review and metanalysis developed in 2019 by Ghasemiesfe et al. (2019) tried to find out the association between marijuana use and cancer development in adults with at least 1 year exposure. Low-strength evidence suggests that more than 10 years of cannabis smoking is associated with an increased risk of developing testicular germ cell tumor. However, its association with other cancers, such as lung and head and neck cancers are of poor quality and inconclusive and limited by low exposure and duration of follow-up.

## 5 Modes of administration

Oral administration of herbal Cannabis has a lower bioavailability (5%–20%) than inhalation, due to the gastric degradation of cannabinoids and first-pass hepatic metabolism (Le Boisselier et al., 2017). The pharmacological effects after oral administration range from 30 min to 3 h and the maximum concentration of cannabinoids in the blood is usually reached within 2 h (Maida and Daeninck, 2016; Brunetti et al., 2020).

Cannabis administration by inhalation seems to be better tolerated and with more predictable effects than orally (Romero-Sandoval et al., 2017). Administration of medicinal cannabis by inhalation can be done in two ways, by vaporization or by smoking, “cannabis vaping” gaining the most popularity in recent years because it eliminates inhalation of smoke by-products such as tar (Ghasemiesfe et al., 2019). Recent studies have shown that vaping products could lead to lung injury, often referred to with the acronym EVALI and is predominately associated with cannabis products (Boyd et al., 2021). Ghasemiesfe & collab showed in a recent meta-analysis that short-term cannabis vaporization has a minimal impact on pulmonary function, however the data regarding long-term exposure is still insufficient and conflicting (Ghasemiesfe et al., 2019).

As for medical cannabis decoctions, and cannabis extract in oil, there are no specific guidelines for a standardized method of preparation provided by any European or international pharmacopoeias. When refrigerated for several days, the cannabinoid concentration decreases, so that extemporaneous preparation is recommended, therefore there are no industrial products available (Hazekamp et al., 2007).

Again, no European or international pharmacopoeia offers a standardized method for preparing cannabis extract in oil. In general, manufacturers extract CBD or THC from the C. Sativa plant, then dilute it with a nontoxic oil. At the moment, there are no FDA or EMA approved cannabinoid oil for treating symptoms associated to cancer, however Epidiolex® it is approved for Lennox-Gastaut and Dravet syndrome (Sekar and Pack, 2019).

Topical administration does not allow the entering of the compounds into the bloodstream, thus considered nonpsychoactive. Their onset of action is 15–30 min after application and effects can last for 3–6 h. Transdermals include patches and gels, have a similar bioavailability as topicals, but can be absorbed into the bloodstream, however, can be time-realeased (Bruni et al., 2018).

## 6 Side-effects

Although there is increasing evidence for the therapeutic potential of the cannabinoids in oncology, a clinician should always be aware of the potential side effects associated. Literature available has a significant level of uncertainty regarding the safety of cannabinoids. This is not a result of the lack of research, but rather caused by the extreme variability in study methodology and quality.

Some of the cannabinoids’ adverse effects are summarized in Table 3.

**TABLE 3:** Common clinical side effects associated to cannabinoid consumption.

Effect on cardiovascular system	• Tachycardia, increased blood pressure, systemic vasodilatation and increased cardiac labor in a dose-dependent manner (Ravi et al., 2018)
	• myocardial infarction, cardiomyopathy, and sudden cardiac death for patients with a history of cardiac diseases (Jouanjus et al., 2011; Frost et al., 2013; Thomas et al., 2014; Tait et al., 2016)
Effect on respiratory system	• Inflammation of large airways, increase airway resistance, and injure lung tissue (Sachs et al., 2015; Joshi et al., 2022)
	• Chronic bronchitis or infections associated with the respiratory tract (Hancox et al., 2010; Tashkin and Tan, 2022)
Psychiatric conditions	• An increased risk of psychotic disorders following acute and repeated consumption in naive users (Ganesh and D’Souza, 2022; Di Forti et al., 2015; Arseneault et al., 2002; Grotenhermen and Müller-Vahl, 2012)
	• Mood disturbances, mania, psychosis and schizophrenia in case of chronic use (Johnson et al., 2021; Ganesh and D’Souza, 2022; Gibbs et al., 2015)
	• Addiction (Connor et al., 2021)
Cognitive and CNS alterations	• Impairment of a wide range of cognitive functions in a dose-relation manner (Broyd et al., 2016; Lorenzetti et al., 2016; Wieghorst et al., 2022)
	• Long-term brain functional and structural alterations (Cousijn et al., 2012; Broyd et al., 2016; Meier et al., 2022)
	• Sedation (Lynch and Campbell, 2011; Borgelt et al., 2013; Kuhathasan et al., 2019)

Cannabinoids can have cardiovascular effects in a dose dependent manner, which can be managed through symptomatic treatment, without the need for hospitalization of the young patient (Ravi et al., 2018). However, senior patients with a history of heart diseases can develop life-threatening cardiovascular side effects, such as myocardial infarction (Tait et al., 2016). The main route of administration of illicitly obtained cannabis is through smoking; obviously, it is not without adverse effects on the respiratory tract.

Regarding safety in pregnancy, it is recommended to avoid the administration, given the possibility of increased risk for stillbirth, preterm birth, fetal growth restriction, miscarriage, and adverse neurodevelopmental consequences. However, much of the existing research was performed in the 1980s, when quantities of THC were lower and the frequency of use was less (Thompson et al., 2019).

Another concern regarding cannabis uses it is about the effects on cognition. It seems it can vary depending on the frequency of use and cumulative dose of the compound. The majority are relatively short lived and diminish over time with abstinence. However, the risk of long-term exposure effects of cannabis use appears to increase with earlier age of onset. Bolla et al. (2002) found in their study that dose-dependent neurocognitive impairment persisted after 28 days of abstinence in heavy young users. Furthermore, there is also evidence that adults who initiated regular cannabis use in adolescence may have structural and functional alterations of the CNS (Wieghorst et al., 2022).

Another important aspect of cannabinoid use is the increasing body of evidence aimed at the adverse effects that they may have causing or precipitating existing psychiatric disorders, highly dependent on dosage, time, potency and patient’s age (Johnson et al., 2021). Several studies suggest that acute cannabis exposure may induce temporary psychosis. Regarding chronic consumption, special attention must be paid to patients diagnosed with psychiatric conditions, because cannabis may exacerbate pre-existing symptoms of psychosis and schizophrenia (Ganesh and D’Souza, 2022; Gibbs et al., 2015).

Any health effects of increased potency cannabinoids depend on whether patients are able and willing to titrate their dose of THC. This may also vary with the experience of users. Among naive users, higher THC content may increase the likelihood of adverse psychological effects, while increasing the risk of dependence and psychotic symptoms if regular users do not titrate their dose. However, there is some evidence that cannabis with a high CBD and low THC content may decrease the risk of psychosis (Mechoulam et al., 2007). In the case of the general population, cannabis use may trigger psychosis and schizophrenia in cases of a predisposition or genetic susceptibility to mental illness, again, depending on the factors previously listed (Grotenhermen, 2007). This risk appears especially in the case of young people, also demonstrated by Andréasson et al. (1987) where 50,465 Swedish male conscripts reported that those who had tried cannabis by age 18 years were 2–4 times more likely to be diagnosed with schizophrenia than those who had not.

One of the main concerns that leads to a reserved attitude regarding the medicinal use of cannabis, both on the side of patients and doctors, is represented by the risk of addiction. Approximately 17% of adolescent users develop addiction, however after 25 years old, the addiction pattern follows a downward trajectory, being rarely addictive (Shekhar, 2014). Data between the 70s–90s have shown some correlation between cannabis and other illicit drug use. Adolescents with addictive behavior (as in use of “heavy drugs”, alcohol, tobacco) were more likely to develop addiction following cannabis consumption. The possible explanation for this was believed to be the opportunity to procure cannabis and other illicit drugs from the same black market or the fact that young consumers have a predisposition for combining cannabis with other substances (Mechoulam et al., 2007).

However, it is important for oncologists to control patients by measures of medical history (vulnerability to mental illness and addiction), and periodic clinical exams, with attention to the proportions of active molecules for the desired clinical effects, to prevent undesired psychoactive effects.

## 7 Conclusion

Cannabinoids have been used as an almost universal remedy for millennia, but their immense inter-strain variability, combined with a high potential of abuse have hindered research until recently. As more countries legalize or decriminalize cannabis use, patients with chronic conditions, especially cancer patients are more and more interested in using these drugs as palliative or curative treatment. Despite this trend, except for their effect on nausea and vomiting cannabinoids are not currently approved in any cancer-related indication. One of the major issues when interpreting available real-world data and translating them in clinical practice is the lack of good practice standards, which are essential especially for this compound. Still, cannabinoids do appear to have some effect in cancer-related pain, especially as co-analgesics and if started early during treatment, and overall seem to improve patient wellbeing by slightly improving different cancer-related symptoms such as mood, appetite or anxiety. The anti-tumor effect of cannabinoids is also currently a matter of debate, and these drugs seem to act as a double-edged sword in relationship to cancer progression due to the plethora of effects of the endocannabinoid system. However, most current data seem to favor the anti-tumor effect of cannabinoids, suggesting they could be used in the future in conjunction with some anti-cancer systemic therapies with the notable exception of immunotherapy.

However, before prescribing, clinicians should take into consideration the profile of the patient to which prescription is made. Young patients, pregnant, with a history of mental health or substance abuse, or elderly with a history of severe cardiovascular diseases should avoid the use of cannabinoids, until more data regarding the safety are available. Another important aspect is the ability of the clinician to choose the right combination of active molecules for the desired effect, the dosage and form of administration, to prevent undesired effects.

Still, a lot of additional data regarding their mechanism of action, posology, interactions with other drugs, or assessing adverse effects is required to access the full potential of these drugs, along with high quality clinical trials, so that oncologists would be more confident when prescribing them to the patients.
